# Purification of functional human ES and iPSC-derived midbrain dopaminergic progenitors using LRTM1

**DOI:** 10.1038/ncomms13097

**Published:** 2016-10-14

**Authors:** Bumpei Samata, Daisuke Doi, Kaneyasu Nishimura, Tetsuhiro Kikuchi, Akira Watanabe, Yoshimasa Sakamoto, Jungo Kakuta, Yuichi Ono, Jun Takahashi

**Affiliations:** 1 Department of Clinical Application, Center for iPS Cell Research and Application, Kyoto University, Sakyo-ku, Kyoto 606-8507, Japan; 2 Department of Life Science Frontiers, Center for iPS Cell Research and Application, Kyoto University, Kyoto 606-8507, Japan; 3 Group for Antibody Engineering, KAN Research Institute Inc, Kobe 650-0047, Japan; 4 Group for Seed Biologics, KAN Research Institute Inc., Kobe 650-0047, Japan; 5 Group for Neuronal Differentiation and Development, KAN Research Institute Inc., Kobe 650-0047, Japan; 6 Group for Regenerative Medicine, KAN Research Institute Inc., Kobe 650-0047, Japan; 7 Department of Neurosurgery, Kyoto University School of Medicine, Kyoto 606-8507, Japan

## Abstract

Human induced pluripotent stem cells (iPSCs) can provide a promising source of midbrain dopaminergic (mDA) neurons for cell replacement therapy for Parkinson's disease (PD). However, iPSC-derived donor cells inevitably contain tumorigenic or inappropriate cells. To eliminate these unwanted cells, cell sorting using antibodies for specific markers such as CORIN or ALCAM has been developed, but neither marker is specific for ventral midbrain. Here we employ a double selection strategy for cells expressing both CORIN and LMX1A::GFP, and report a cell surface marker to enrich mDA progenitors, LRTM1. When transplanted into 6-OHDA-lesioned rats, human iPSC-derived LRTM1^+^ cells survive and differentiate into mDA neurons *in vivo*, resulting in a significant improvement in motor behaviour without tumour formation. In addition, there was marked survival of mDA neurons following transplantation of LRTM1^+^ cells into the brain of an MPTP-treated monkey. Thus, LRTM1 may provide a tool for efficient and safe cell therapy for PD patients.

## Abstract (web summary)

Midbrain dopaminergic neurons generated from stem cells show promise for the treatment of Parkinson's disease. Here, the authors use the cell surface marker, LRTM1, to enrich the midbrain dopaminergic progenitors and show improved motor function/cell survival when grafted into rat/monkey brains, respectively.

## Graphical abstract

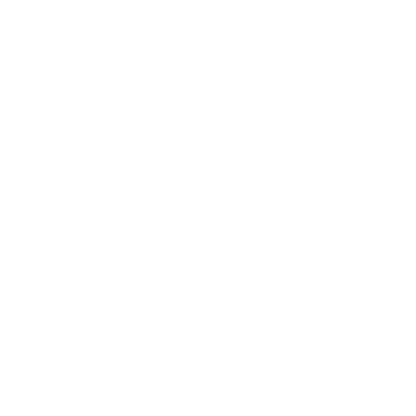

Parkinson's disease (PD) is a progressive neurodegenerative disorder caused by a loss of midbrain dopaminergic (mDA) neurons. Cell replacement therapy using human pluripotent stem cells (PSCs) is expected to ameliorate the disease. A combination of dual SMAD inhibition and GSK3B inhibition has enabled us to induce mDA neurons from human embryonic stem cells (hESCs) and human induced PSCs (hiPSCs)^1^,^2^. However, the differentiated cells are heterogeneous and may contain residual undifferentiated stem cells or proliferating neural progenitor cells, which may cause tumour formation^3^,^4^,^5^,^6^. Furthermore, previous clinical trials using fetal mesencephalic cells suggested that contaminating serotonergic neurons may cause graft-induced dyskinesia^7^,^8^. Therefore, the elimination of these unwanted cells is critical for clinical application in terms of safety.

For that purpose, fluorescence-activated cell sorting (FACS) using antibodies for CORIN, a floor plate (FP) marker^9^, or ALCAM, a central nervous system microvascular endothelium marker^10^, have been developed. However, both CORIN and ALCAM are expressed not only in the ventral midbrain (VM) but also in more caudal FP during early brain development^11^,^12^. Therefore, cell sorting using anti-CORIN or anti-ALCAM antibodies alone cannot exclude the possibility of contaminating cells from outside the VM.

To overcome this problem, one possible strategy is to combine a refined differentiation protocol that induces mesencephalon with FACS using anti-CORIN antibody^9^. Another strategy is double sorting using antibodies for CORIN and OTX2, a transcriptional factor of the forebrain and midbrain^13^. In that study, an OTX2::GFP knock-in (KI) mouse ESC (mESC) line was established and made to differentiate into neurons. CORIN^+^OTX2::GFP^+^ cells gave rise to more mDA neurons than unsorted cells and improved abnormal motor behaviour of 6-hydroxydopamine (6-OHDA)-lesioned rats when grafted into the striatum^13^. Theoretically, the combination of a fore- and midbrain marker and an FP marker enables us to isolate VM cells. However, OTX2 is not a cell surface protein but a transcription factor, meaning that its antibody cannot be used in FACS in clinical settings.

To address this issue, we took advantage of LMX1A, a transcription factor for the midbrain^14^. We produced LMX1A::GFP KI mESC line as a tool for visualizing midbrain cells and then performed microarray analysis to identify cell surface markers specific for VM cells by comparing gene expression profiles between CORIN^+^ and CORIN^−^ cells in LMX1A::GFP^+^ cells. Here we report that a cell surface protein, LRTM1, is specifically expressed in mouse fetal VM. Purification of hiPSC-derived LRTM1^+^ cells resulted in a higher density of mDA neurons surviving in the striatum of 6-OHDA-lesioned rats than did unsorted cells and better behavioural recovery. In addition, hiPSC-derived LRTM1^+^ cells survived well in a short-term primate study without overgrowth.

## Results

### Purification of mDA progenitors from CORIN^+^LMX1A^+^ cells

**Figure 1:**
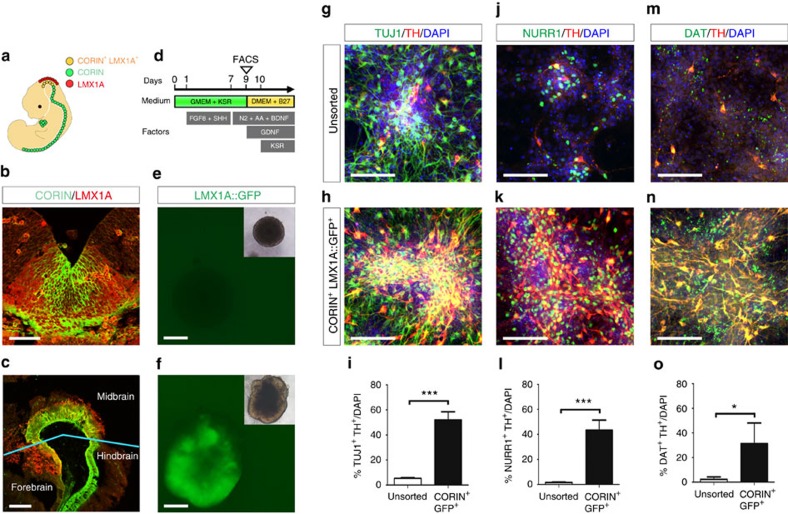
Purification of mDA progenitors by co-expression of CORIN and LMX1A::GFP. (**a**) Schematic diagram of CORIN and LMX1A expression during early development of mouse. (**b**,**c**) Immunohistochemical images for CORIN (green) and LMX1A (red) in coronal and sagittal sections of E11.5 fetal mouse. Scale bars, 50 μm (**b**) and 200 μm (**c**). (**d**) Schematic diagram of neuronal differentiation from mESCs. (**e**,**f**) LMX1A::GFP expression of the serum-free floating culture of embryoid body-like aggregates with the quick reaggregation (SFEBq)-cultured mESC aggregates on day 2 (**e**) and day 9 (**f**). Scale bars, 200 μm. Insets indicate bright-field images of the aggregates. (**g**–**o**) Immunofluorescence images of the cells from unsorted and CORIN^+^LMX1A::GFP^+^ cells for TUJ1 (green), NURR1 (green), DAT (green), TH (red) and 4′, 6′-diamidino-2-phenylindole (DAPI; blue) on day 14. Scale bars, 70 μm. Quantification of TUJ1^+^TH^+^, NURR1^+^TH^+^ and DAT^+^TH^+^ cells in unsorted cells (*n*=4) versus CORIN^+^LMX1A::GFP^+^ cells (*n*=4) on day 14. Asterisks indicate statistical significance as determined by Student's *t*-test, *P<0.05 and ***P<0.001. Error bars indicate s.e.m.

During early development of mouse brain, LMX1A is expressed in midbrain^14^, whereas CORIN is expressed in FP^9^. Based on these findings, we hypothesized that VM cells including mDA progenitors can be purified by a combination of these two markers (Fig. 1a–c). To visualize midbrain cells, we produced an LMX1A::GFP KI mESC line. The cells were induced to differentiate into neuronal lineage by the serum-free floating culture of embryoid body-like aggregates with the quick reaggregation method (Fig. 1d)^15^. Although LMX1A::GFP expression was not detectable in the early differentiation stage (Fig. 1e), it became apparent on day 9 (Fig. 1f). In support of this trend, the messenger RNA levels of *Oct-4* (also known as *Pou5f1*) gradually decreased from day 0 (Supplementary Fig. 1a). The emergence of LMX1A::GFP^+^ cells closely paralleled the onset of not only *Lmx1a*, but also *Corin*, *Nurr1* (also known as *Nr4a2*) and *Th* (Supplementary Fig. 1b–e). A flow cytometric analysis revealed that the percentage of CORIN^+^LMX1A::GFP^+^ cells reached a peak on day 9 (30.5±3.1% of total cells; *n*=3; Supplementary Fig. 1f). Immunostaining of the cells on day 9 showed that NURR1, a marker for postmitotic DA progenitors, was expressed by CORIN^−^LMX1A::GFP^+^ cells (Supplementary Fig. 1g). Intriguingly, FOXA2^+^ FP cells^16^,^17^ were enriched in CORIN^+^LMX1A::GFP^+^ and CORIN^−^LMX1A::GFP^+^ populations on day 9 (Supplementary Fig. 2). Comparative gene expression analysis among these populations revealed that CORIN^+^LMX1A::GFP^+^ cells expressed significantly lower levels of *Six3*, a forebrain marker, and *Gbx2*, a hindbrain marker, compared with CORIN^−^LMX1A::GFP^−^ cells or CORIN^−^LMX1A::GFP^+^ cells (Supplementary Fig. 3a,b). On the other hand, CORIN^+^LMX1A::GFP^+^ cells expressed significantly higher levels of *Corin*, *Lmx1a* and *Nurr1* compared with other populations (Supplementary Fig. 3c–e). To determine whether CORIN^+^LMX1A::GFP^+^ cells give rise to mature mDA neurons more efficiently, we cultured CORIN^+^LMX1A::GFP^+^ cells and unsorted cells for another 5 days for maturation (Fig. 1d). Double-labelled immunostaining revealed that CORIN^+^LMX1A::GFP^+^ cells gave rise to mDA neurons, which expressed TH, NURR1 and dopamine transporter (DAT) (also known as SLC6A3), more frequently than unsorted cells (Fig. 1g–o). These results indicate that mDA progenitors were enriched in the CORIN^+^LMX1A::GFP^+^ population.

### LRTM1 is a cell surface marker for mDA progenitors

**Figure 2:**
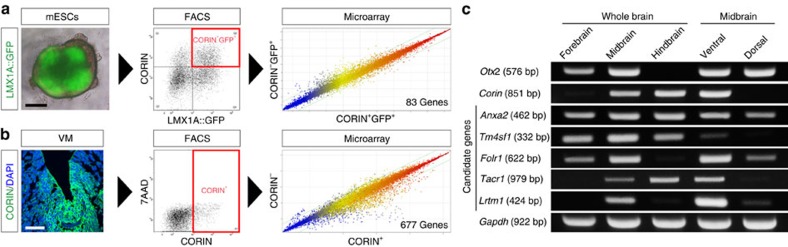
Gene expression profiles between CORIN^+^ and CORIN^−^ populations from mESC-derived LMX1A::GFP^+^ cells or fetal mouse VM. (**a**) Comparison of gene expression profiles between CORIN^+^ and CORIN^−^ cells in LMX1A::GFP^+^ cells on day 9. Scale bars, 250 μm. (**b**) Comparison of gene expression profiles between CORIN^+^ and CORIN^−^ cells in E11.5 fetal mouse VM. Scales bars, 50 μm. (**c**) Semi-quantitative RT–PCR analysis of *Otx2*, *Corin*, *Anxa2*, *Tm4sf1*, *Folr1*, *Tacr1*, *Lrtm1* and *Gapdh* in E11.5 fetal mouse brain.

To identify a cell surface marker of mDA progenitors, we performed microarray analyses to compare gene expression profiles between the following cell populations: (1) mESC-derived CORIN^+^LMX1A::GFP^+^ cells versus CORIN^−^LMX1A::GFP^+^ cells on day 9, based on the finding that the percentage of CORIN^+^LMX1A::GFP^+^ cells peaked on day 9 (Supplementary Fig. 1f); and (2) CORIN^+^ cells versus CORIN^−^ cells in E11.5 mouse fetal VM, based on the finding that CORIN is expressed by actively dividing cells in the ventricular zone of E11.5 VM (ref. 9 and Supplementary Fig. 4). We chose 83 and 677 genes from the first and the second analysis, respectively, which were expressed at higher levels in the CORIN^+^ population (Fig. 2a,b). Among these candidates, 16 genes were commonly upregulated in ESC-derived CORIN^+^LMX1A::GFP^+^ cells and CORIN^+^ cells in fetal mouse VM (Supplementary Data 1). We further selected genes coding a cell surface antigen and conserved in humans, leaving five genes as candidates for a cell surface marker of mDA progenitors: annexin A2 (*Anxa2*), Transmembrane 4 superfamily member 1 (*Tm4sf1*), Folate receptor 1 (*Folr1*), Tachykinin receptor 1 (*Tacr1*) and leucine-rich repeats (LRRs) and transmembrane domains 1 (*Lrtm1*). A semi-quantitative reverse transcriptase–PCR (RT–PCR) analysis revealed that only *Lrtm1* (also known as *A930016D02Rik*) was specifically expressed in E11.5 mouse fetal VM (Fig. 2c).

**Figure 3:**
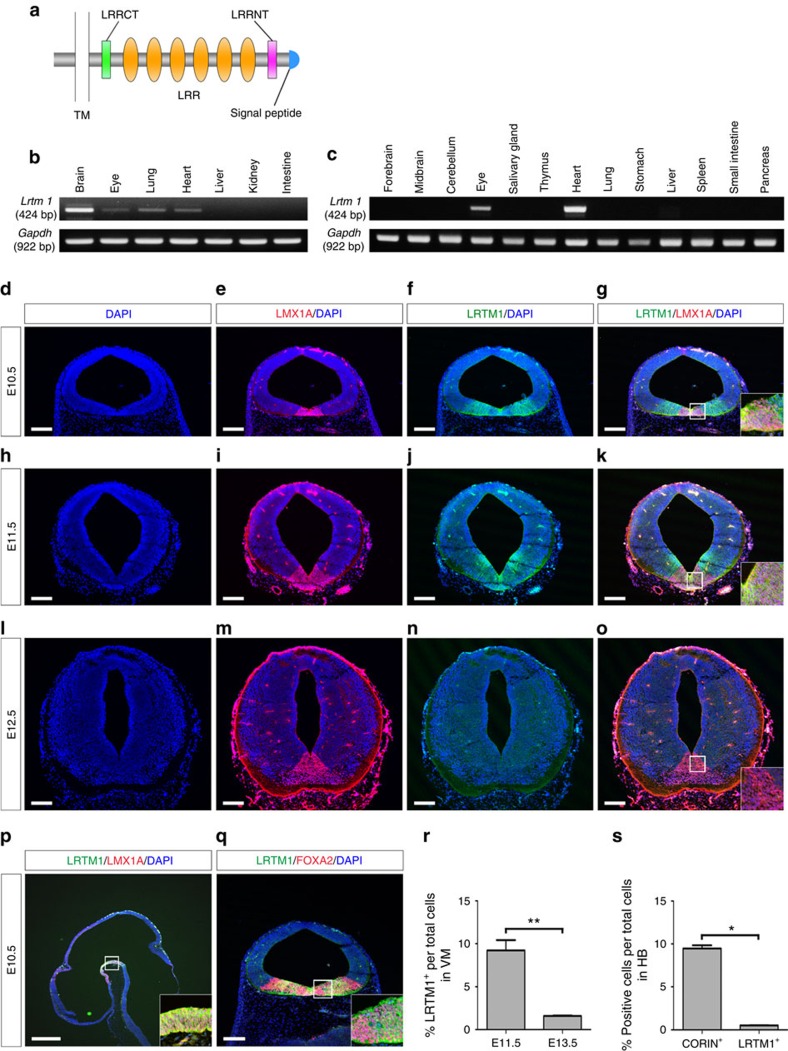
LRTM1 is selectively expressed in VM during early brain development. (**a**) Schematic construction of LRTM1. (**b**) In E11.5 fetal mouse, LRTM1 mRNA was detected in the brain, eye, lung and heart. (**c**) In adult mouse, LRTM1 mRNA was detected in the eye and heart only. (**d**–**o**) Immunohistochemical images of fetal mouse midbrain for 4′, 6′-diamidino-2-phenylindole (DAPI; blue), LMX1A (red) and LRTM1 (green). Insets indicate magnified images of LMX1A^+^ DA progenitors. Scale bars, 150 μm. (**p**) Immunohistochemical image of E10.5 fetal mouse for LRTM1 (green), LMX1A (red) and DAPI (blue) in the sagittal section. Inset indicates magnified images of LMX1A^+^ DA progenitors. Scale bar, 400 μm. (**q**) Immunohistochemical image of E10.5 fetal mouse for LRTM1 (green), FOXA2 (red) and DAPI (blue) in the coronal section. Inset indicates magnified images of FOXA2^+^ FP cells. Scale bar, 150 μm. (**r**) Quantification of LRTM1^+^ cells in E11.5 VM (*n*=4) versus E13.5 VM (*n*=4). (**s**) Quantification of immunoreactive cells in the hindbrain stained for anti-Corin (*n*=4) versus anti-LRTM1 antibodies (*n*=4). Asterisks indicate statistical significance as determined by Student's *t*-test, *P<0.05 and **P<0.01. Error bars indicate s.e.m.

LRTM1 belongs to the extracellular LRRs superfamily^18^. It is composed of a signal peptide, LRR amino terminus, six LRRs, an LRR carboxy terminus, a transmembrane domain and a short cytoplasmic tail containing a short stretch of acidic residues (Fig. 3a)^18^. As little is known about this protein, we examined the expression of LRTM1 in mouse. A semi-quantitative RT–PCR analysis revealed that LRTM1 mRNA was highly expressed in the brain but weakly expressed in the eye, lung and heart of E11.5 fetal mouse (Fig. 3b). In adult mouse, LRTM1 was expressed only in the eye and heart (Fig. 3c). A double-labelled immunofluorescence study showed that the expression of LRTM1 was restricted to the VM during E10.5 to E11.5. Its expression area was overlapped with that of LMX1A, but LRTM1 was more laterally expressed (Fig. 3d–k,p). On E12.5, a stage when mature DA neurons are generated, the expression of LRTM1 disappeared, whereas LMX1A was still expressed (Fig. 3l–o). In E10.5, the expression of LRTM1 was also overlapped with that of FOXA2, an FP marker (Fig. 3q).

In support of these results, a flow cytometric analysis revealed that LRTM1^+^ cells were more abundantly contained in mouse VM on E11.5 than on E13.5 (Fig. 3r). Furthermore, less LRTM1^+^ cells were contained in E11.5 mouse hindbrain compared with CORIN^+^ cells (Fig. 3s).

These results indicate that the expression of LRTM1 in the developing brain is restricted in terms of the developmental stage, the time of emergence of mDA progenitors and location, namely the VM.

### Enrichment of mouse mDA progenitors by LRTM1 sorting

To determine whether sorting with anti-LRTM1 antibody contributes to the enrichment of mDA progenitors, we dissociated E11.5 mouse VM tissue and purified LRTM1^+^ cells by FACS. The LRTM1^+^ population contained more FOXA2^+^LMX1A^+^ mDA progenitors compared with an unsorted population (42.6±1.7% versus 11.1±1.6%; *n*=4 for each group; Supplementary Fig. 5).

When we induced DA differentiation by the serum-free floating culture of embryoid body-like aggregates with the quick reaggregation method, transient expression of *Lrtm1* was observed in mESC/iPSC lines (Supplementary Fig. 6a). At 9 days after differentiation of the mouse iPSC (miPSC) line 440A3, we found that ∼10% of total cells were LRTM1^+^ and purified them by FACS. These cells contained more FOXA2^+^LMX1A^+^ mDA progenitors compared with unsorted cells (77.2±2.1% versus 42.5±1.6%; *n*=5 and 6, respectively; Supplementary Fig. 6b–d).

To determine whether miPSC-derived LRTM1^+^ cells can survive *in vivo*, we purified the LRTM1^+^ cells by FACS on day 9 and 2 days later we injected them into the striatum of 6-OHDA-lesioned rats. A large number of TH^+^ neurons survived (1,922±691 cells per graft; *n*=3) and extended their neurites into the host striatum at 6 weeks after transplantation (Supplementary Fig. 6e).

These results indicate that mDA progenitors can be isolated from mouse embryo and mouse PSCs by FACS using anti-LRTM1 antibody.

### Enrichment of human mDA progenitors by LRTM1 sorting

**Figure 4:**
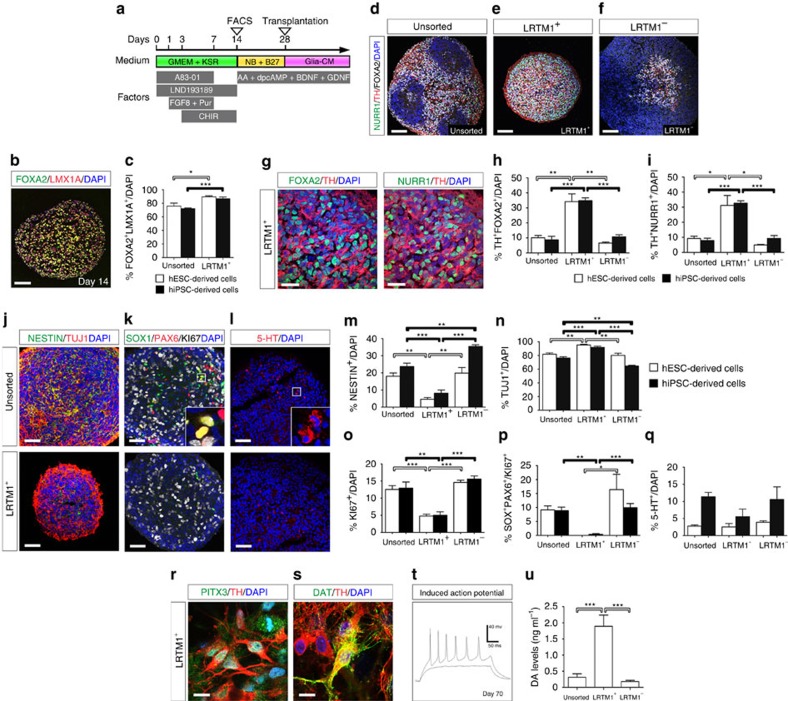
Human LRTM1^+^ cells generate mature mDA neurons *in vitro*. (**a**) Diagram of neuronal differentiation from human PSCs (hPSCs). (**b**) Immunofluorescence image of a hESC-derived LRTM1^+^ sphere on day 14 for FOXA2 (green), LMX1A (red) and 4′, 6′-diamidino-2-phenylindole (DAPI; blue). Scale bars, 100 μm. (**c**) Quantification of FOXA2^+^LMX1A^+^ cells in unsorted cells versus LRTM1^+^ cells on day 14 (hESC: *n*=6; hiPSC: *n*=6). (**d**–**f**) Immunofluorescence images of spheres from unsorted cells, LRTM1^+^ cells and LRTM1^−^ cells on day 28 for NURR1 (green), TH (red), FOXA2 (white) and DAPI (blue). Scale bars, 100 μm. (**g**) Immunofluorescence images of a sphere from LRTM1^+^ cells for FOXA2 (green), NURR1 (green), TH (red) and DAPI (blue) on day 28. Scale bars, 50 μm. Quantification of TH^+^FOXA2^+^ (**h**) and TH^+^NURR1^+^ cells (**i**) in unsorted cells (hESC: *n*=4; hiPSC: *n*=3) versus LRTM1^+^ cells (hESC: *n*=4; hiPSC: *n*=3) versus LRTM1^−^ cells (hESC: *n*=3; hiPSC: *n*=3). (**j**–**l**) Immunofluorescence images of spheres from unsorted and LRTM1^+^ cells for NESTIN (green), SOX1 (green), TUJ1 (red), PAX6 (red), 5-HT (red), KI67 (white) and DAPI (blue) on day 28. Scale bars, 50 μm. Quantification of NESTIN^+^ (**m**), TUJ1^+^ (**n**), KI67^+^ (**o**), SOX1^+^PAX6^+^ (**p**) and 5-HT^+^ cells (**q**) in unsorted cells (hESC: *n*=5; hiPSC: *n*=4) versus LRTM1^+^ cells (hESC: *n*=5; hiPSC: *n*=4) versus LRTM1^−^ cells (hESC: *n*=5; hiPSC: *n*=4). (**r**,**s**) Immunofluorescence images of LRTM1^+^ cells for PITX3 (green), DAT (green), TH (red) and DAPI (blue) on day 70. Scale bars, 10 μm. (**t**) Current clamp recordings of induced action potentials by brief current pulses from human ES-derived DA neurons on day 70. (**u**) Levels of DA in hESC-derived unsorted (*n*=5) versus LRTM1^+^ (*n*=5) versus LRTM1^−^ (*n*=5) cultures on day 42. Asterisks indicate statistical significance as determined by Student's *t*-test, *P<0.05 and ***P<0.001 (**c**), and a one-way analysis of variance with Bonferroni's multiple comparison test, *P<0.05, **P<0.01 and ***P<0.001 (**h**,**i**,**m**–**p**,**u**). Error bars indicate s.e.m.

To determine whether the same strategy can be applied to human mDA progenitors, we induced DA neurons from hESCs (Kh-ES1; ref. 19) and hiPSCs (1039A1; ref. 20) as previously reported (Fig. 4a)^11^. A comparative temporal gene expression analysis revealed that the expression of a pluripotent cell marker (*OCT4*) gradually decreased, whereas that of a basal and FP marker (*FOXA2*) and a midbrain marker (*LMX1A*) reached a plateau on days 7–14 (Supplementary Fig. 7a–c). Markers for more mature DA neurons (*NURR1* and *TH*) gradually increased for 35 days and the expression of *LRTM1* peaked on day 14 (Supplementary Fig. 7d–f).

Based on these results, we purified human PSC-derived LRTM1^+^ cells on day 14. An immunofluorescence study of the spheres 12 h after sorting revealed that FOXA2^+^LMX1A^+^ mDA progenitors were more abundantly contained in LRTM1^+^ populations compared with unsorted ones (hESC: 89.5±1.5% versus 75.9±4.5%; hiPSC: 86.7±2.6% versus 72.0±1.3%; *n*=6 for each group; Fig. 4b,c). We continued culture of the spheres and performed triple-labelled immunostaining on day 28. Midbrain DA neurons expressing TH, FOXA2 and NURR1 were more frequently observed in LRTM1^+^ populations compared with unsorted and LRTM1^−^ populations (Fig. 4d–i). Further analysis of the spheres revealed that neurons expressing TUJ1 were more frequently observed in the LRTM1^+^ population compared with the unsorted and LRTM1^−^ populations, whereas neural precursor cells expressing NESTIN were more frequently observed in the unsorted and LRTM1^−^ populations (Fig. 4j,m,n). We recently reported that in a transplantation of iPSC-derived early neural cells, SOX1^+^PAX6^+^KI67^+^ cells form rosettes in the grafts and contribute to graft expansion^21^. In the present work, we found that the percentage of KI67^+^ proliferating cells was significantly lower and SOX1^+^PAX6^+^KI67^+^ cells were almost eliminated in the LRTM1^+^ population (Fig. 4k,o,p). There were no PSCs expressing OCT-4 and only few serotonergic neurons expressing 5-HT in the spheres (Fig. 4l,q). On day 70, the LRTM1^+^ cells expressed PITX3, a marker for mature mDA neurons^22^,^23^, and DAT, a principal regulator of DA neurotransmission (Fig. 4r,s). These cells exhibited action potentials according to electrophysiological analysis (Fig. 4t). DA levels in LRTM1^+^ cell cultures were at least six times higher than in unsorted and LRTM1^−^ cell cultures on day 42 (1.9±0.3, 0.3±0.1 and 0.2±0.0 pg ml^−1^; *n*=5 for each group; Fig. 4u). These results indicate that mDA progenitors can be isolated from human PSCs by FACS using anti-LRTM1 antibody, and that LRTM1^+^ cells differentiate into mature mDA neurons *in vitro*.

### Human LRTM1^+^ cells differentiated into mDA neurons *in vivo*

**Figure 5:**
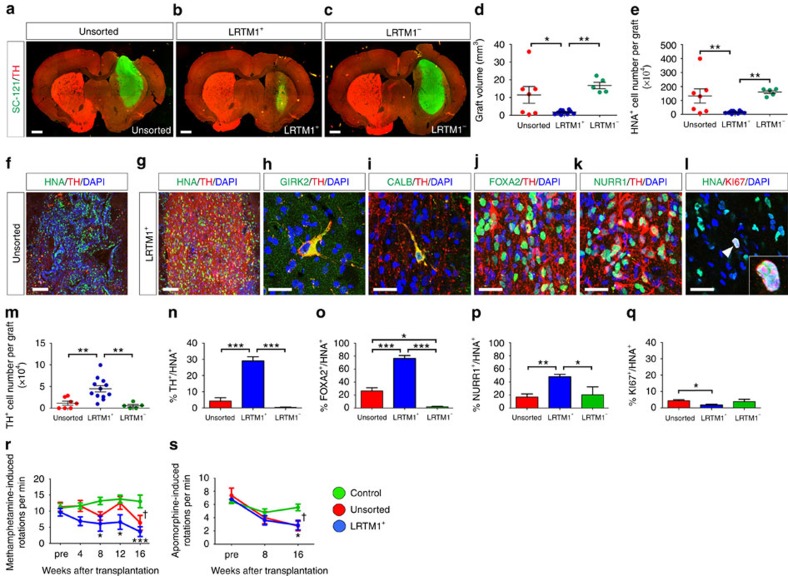
Human LRTM1^+^ cells generate functional DA neurons *in vivo* following transplantation. (**a**–**c**) Immunofluorescence images of the graft for SC-121 (green) and TH (red) at 12 weeks. Scale bars, 1 mm. (**d**,**e**) Quantification of graft volumes and HNA^+^ cells in unsorted cells (*n*=7) versus LRTM1^+^ cells (*n*=12) versus LRTM1^−^ cells (*n*=5) at 12 weeks. Immunofluorescence images of a graft containing unsorted cells (**f**) and LRTM1^+^ cells (**g**) for 4′, 6′-diamidino-2-phenylindole (DAPI; blue), HNA (green) and TH (red) at 12 weeks. Scale bars, 100 μm. (**h**–**l**) Immunofluorescence images of a graft containing LRTM1^+^ cells for GIRK2 (green), CALBINDIN (green), FOXA2 (green), NURR1 (green), HNA (green), TH (red), KI67 (red) and DAPI (blue) at 12 weeks. Scale bars, 25 μm. Quantification of TH^+^ (**m**), TH^+^HNA^+^ (**n**), FOXA2^+^HNA^+^ (**o**), NURR1^+^HNA^+^ (**p**) and KI67^+^HNA^+^ (**q**) cells in unsorted cells (*n*=7) versus LRTM1^+^ cells (*n*=12) versus LRTM1^−^ cells (*n*=5) at 12 weeks. Asterisks indicate statistical significance as determined by a one-way analysis of variance (ANOVA) with Bonferroni's multiple comparison test, *P<0.05, **P<0.01 and ***P<0.001. Error bars indicate s.e.m. (**r**,**s**) Quantification of motor behavior of 6-OHDA-lesioned rats for 16 weeks posttransplantation. (**r**) Methamphetamine-induced rotation (control: *n*=8; unsorted: *n*=7; LRTM1^+^: *n*=7) was performed every 4 weeks after transplantation. (**s**) Apomorphine-induced rotation was performed every 8 weeks after transplantation (control: *n*=7; unsorted: *n*=7; LRTM1^+^: *n*=7). Asterisks indicate statistical significance between control and LRTM1^+^ as determined by a two-way ANOVA with Bonferroni's multiple comparison test, *P<0.05 and ***P<0.001. Dagger indicates statistical significance between control and unsorted as determined by a two-way ANOVA with Bonferroni's multiple comparison test, ^†^*P*<0.05.

To investigate the survival and proliferation of LRTM1^+^ cells *in vivo*, we transplanted unsorted, LRTM1^+^ and LRTM1^−^ cells (1.3 × 10^5^ cells in 2 μl for each condition) into the striatum of 6-OHDA-lesioned rats on day 28. Immunostaining for SC-121, a human cytoplasmic marker, at 12 weeks revealed that the grafted cells survived in the rat brain. The size of the LRTM1^+^ cell-derived graft was significantly smaller (Fig. 5a–d) and consistently the number of surviving human cells (HNA^+^ cells) was significantly lower than that of unsorted cells and LRTM1^−^ cells (Fig. 5e). In spite of these observations, the number of TH^+^ DA neurons was higher in the LRTM1^+^ cell-derived grafts compared with those of unsorted or LRTM1^−^ cells (44,777±7,203 versus 11,504±4,689 versus 5,632±2,919 cells; *n*=12, 7 and 5, respectively; Fig. 5f,g,m). Furthermore, LRTM1^+^ cell-derived grafts extended neuronal fibres more extensively into the host brain (Supplementary Fig. 8). Accordingly, the percentage of DA neurons among surviving human cells was highest in the LRTM1^+^ cell-derived grafts (29.0±2.6% versus 4.2±2.1% versus 0.3±0.2%, *n*=12, 7 and 5, respectively; Fig. 5n). In addition, 58.3 and 32.4% of these neurons co-expressed GIRK2 (also known as KCNJ6), an A9 DA neuron marker (Fig. 5h), or CALBINDIN, an A10 DA neuron marker (Fig. 5i). To elucidate graft components other than the mature mDA neurons, we performed immunostaining to detect early mDA progenitors (FOXA2; Fig. 5j) and postmitotic mDA progenitors (NURR1; Fig. 5k). The percentages of FOXA2^+^ and NURR1^+^ cells among surviving human cells were highest in the LRTM1^+^ cell-derived grafts compared with those derived from unsorted and LRTM1^−^ cells (FOXA2^+^/HNA^+^: 76.4±4.6% versus 26.4±4.9% versus 2.0±0.6%; NURR1^+^/HNA^+^: 48.1±3.7% versus 17.1±4.5% versus 20.3±12.2%; *n*=12, 7 and 5, respectively; Fig. 5o,p). Taken together, in the LRTM1^+^ cell-derived grafts, ∼80% of the cells were in the lineage of mDA neurons, in which 30% became mature mDA neurons and the remaining 70% were still in the stage of progenitors. We also found a small amount of GFAP^+^HNA^+^ cells in the grafts, indicating the presence of donor-derived astrocytes (Supplementary Fig. 9). Proliferating human cells were fewer in the LRTM1^+^ cell-derived grafts (1.8±0.5% versus 4.3±0.6% versus 3.8±1.4%, *n*=12, 7 and 5, respectively; Fig. 5l,q), in which most of the KI67^+^ cells also expressed FOXA2, indicating that they were early mDA progenitors (Supplementary Fig. 9). 5-HT^+^ serotonergic neurons were hardly observed in the grafts (<0.3%).

Next, to investigate the function of LRTM1^+^ cells *in vivo*, we transplanted unsorted and LRTM1^+^ cells (1.3 × 10^5^ cells in 2 μl for each condition) into the striatum of 6-OHDA-lesioned rats on day 28. Behavioural analysis showed significant motor improvement of apomorphine- and methamphetamine-induced rotational asymmetry in both the unsorted and LRTM1^+^ groups at 16 weeks after transplantation (Fig. 5r,s). An immunofluorescence study of these rats revealed that the number of TH^+^ DA neurons was higher in the LRTM1^+^ cell-derived grafts compared with those derived from unsorted cells (11,702±2,566 cells versus 1,102±349 cells; *n*=7 for each group; Supplementary Fig. 10). However, there was no clear correlation between the number of TH^+^ neurons and the behavioural recovery (Supplementary Fig. 10b).

**Figure 6:**
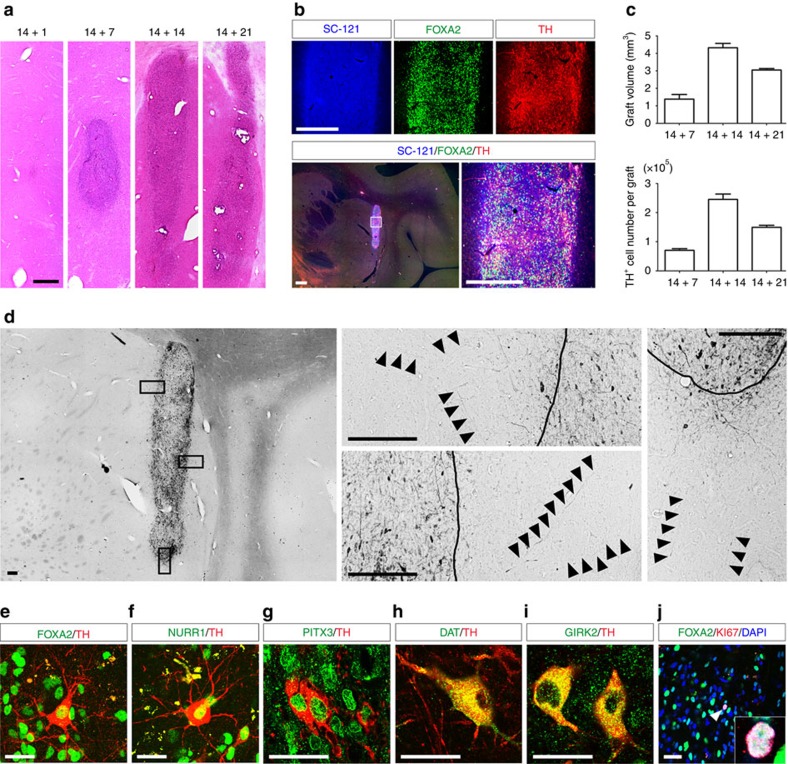
HiPSC-derived LRTM1^+^ cells survived and differentiated into mature DA neurons in primate PD model. (**a**) HE staining of the grafts containing LRTM1^+^ cells at 12 weeks after transplantation. Scale bar, 500 μm. (**b**) Immunofluorescence images of the graft derived from day 28 LRTM1^+^ cells for SC-121 (blue), FOXA2 (green) and TH (red) at 12 weeks after transplantation. Scale bars, 500 μm. (**c**) Quantification of graft volume and TH^+^ cells in the grafts derived from day 21, day 28 and day 35 LRTM1^+^ cells at 12 weeks after transplantation (*n*=2 for each day). (**d**) DAB staining of a graft derived from day 28 LRTM1^+^ cells for TH at 12 weeks after transplantation. Scale bars, 200 μm. (**e**–**j**) Immunofluorescence images of a graft containing LRTM1^+^ cells for FOXA2 (green), NURR1 (green), PITX3 (green), DAT (green), GIRK2 (green), TH (red), KI67 (red) and 4′, 6′-diamidino-2-phenylindole (DAPI; blue) at 12 weeks after transplantation. Scale bars, 30 μm (**e**, **f**), 15 μm (**g**–**i**) and 50 μm (**j**).

Finally, we examined the survival and differentiation of hiPSC-derived LRTM1^+^ cells in the brain of 1-methyl-4-phenyl-1,2,3,6-tetrahydropyridine (MPTP)-treated cynomolgus monkeys. LRTM1^+^ cells were sorted on day 14 and the cultured spheres (1.0 × 10^6^ cells in 4 μl) were injected into the putamen on day 15, 21, 28 or 35. Three months after transplantation, the grafted cells were recognized by hematoxylin and eosin (HE)-staining and immunostaining for SC-121 (Fig. 6a,b). In the case of the day 15 cells (1 day after cell sorting), only few cells survived in the brain. Among the other conditions, the largest number (∼2.4 × 10^5^) of TH^+^ cells was observed in the grafts derived from day 28 cells (Fig. 6c) and these cells extended TH^+^ neuronal fibres into the host brain (Fig. 6d). Furthermore, most of them co-expressed FOXA2, NURR1 and PITX3 (Fig. 6e–g), and some of these cells, were large in size and expressed DAT (Fig. 6h) and GIRK2 (Fig. 6i), indicating they were A9 mDA neurons. A small amount (<0.5%) of the cells still expressed KI67^+^, which, similar to the above, also expressed FOXA2 (Fig. 6j), indicating they were early mDA progenitors. No 5-HT^+^ serotonergic neurons were observed in the graft. These results suggested that hiPSC-derived LRTM1^+^ cells efficiently survived and differentiated into mature mDA neurons in primate brain.

## Discussion

In this study, we employed a double selection strategy for cells expressing both CORIN and LMX1A::GFP, and report a novel cell surface marker for mDA progenitors, LRTM1. Compared with unsorted cells, hiPSC-derived LRTM1^+^ cells differentiated into mDA neurons more efficiently *in vitro*. When grafted into the brains of 6-OHDA-lesioned rats and an MPTP-treated monkey, the sorted LRTM1^+^ cells differentiated into functional mDA neurons and contained few proliferating or serotonergic cells.

LRTM1 belongs to the extracellular LRRs superfamily^18^. LRRs are highly versatile and evolvable protein–ligand interaction motifs found in a large number of proteins with diverse functions. Especially those with extracellular LRRs are involved in various aspects of nervous system development, including axon guidance, target selection and synapse formation^24^,^25^. LRTM1 has a predicted PSD-95/Discs-large/ZO-1 domain-binding sequence at its C terminus, suggestive of synaptic location^18^. LRTM1 is similar to LRRTM1, which has ten extracellular LRRs and is expressed in mouse brain during early development^26^. LRRTM1 also contains a PSD-95/Discs-large/ZO-1 domain-binding sequence at its C terminus^25^. Furthermore, it binds to the presynaptic adhesion molecule neurexin^27^,^28^,^29^, suggesting a role in synapse formation.

A previous study using transgenic mESC reporter lines concluded that the NURR1^*+*^ stage is best for the survival of ESC-derived DA neurons^30^, whereas other reports have shown that DA progenitors are enriched by sorting cells that express CORIN^11^ or ALCAM^12^. NURR1 is a transcription factor expressed by postmitotic mDA progenitors in the intermediate and mantle zones of the developing VM and also by mature mDA neurons^31^,^32^. On the other hand, CORIN is expressed by earlier mDA progenitors in the ventricular zone of the developing VM^9^,^32^. Consistent with these previous reports, we confirmed that NURR1 was expressed by CORIN^−^LMX1A::GFP^+^ cells (Supplementary Fig. 1g) in the differentiated LMX1A::GFP KI ES cells on day 9. These results suggest that early mDA progenitors can be sorted by using anti CORIN and LRTM1 antibodies. Both CORIN and ALCAM were expressed not only in the VM, but also in the caudal FP in the E11.5 mouse brain (Supplementary Fig. 4). In addition, ALCAM was also expressed in dorsal midbrain. In contrast, the expression of LRTM1 was restricted to the VM (Fig. 3d-s). More importantly, the expression was observed only during E10.5 and E11.5, which is when DA progenitors emerge in the VM^12^,^33^,^34^. ALCAM was identified by microarray analysis using E12.5 mouse brain^12^, which was when the expression of LRTM1 almost disappeared (Fig. 3n). These findings indicate that LRTM1 is a more selective marker for early mDA progenitors in terms of time and localization.

In a behavioural evaluation using 6-OHDA-lesioned rats, significant improvement of abnormal behaviour was observed in rats that received either unsorted and cultured LRTM1^+^ grafts (Fig. 5r,s). This finding is consistent with ours^11^ and other reports^1^,^2^,^35^,^36^. An immunofluorescence study of the rats after the behavioural analysis revealed that the number of TH^+^ DA neurons in the grafts of unsorted cells was 1,102±349. Consistently, a previous study showed that <500 surviving TH^+^ cells derived from hESCs are sufficient to exert a behavioural effect^35^. In this study, we could not observe a clear correlation between the number of TH^+^ neurons and the behavioural recovery in a methamphetamine-induced rotational analysis. However, it was revealed that even 319 TH^+^ cells could lead to the behavioural recovery of 6-OHDA-lesioned rats (Supplementary Fig. 10), but this was not true for other rats. These results suggest that the behavioural recovery can be attributed not only to the number of surviving TH^+^ neurons but also possibly to the quality of TH^+^ neurons, neurite extensions from the grafts and the condition of the host environment. Furthermore, there was a large difference in the number of surviving TH^+^ neurons between 12 and 16 weeks posttransplantation. This difference might be due to the different timing of killing (12 versus 16 weeks) or variability in the donor cells such as their differentiation stage and viability in each cell preparation. In the LRTM1^+^ cell-derived grafts, the percentage of TH^+^ DA neurons per total surviving cells at 12 weeks was 29.0±2.6%, which was larger than those of CORIN^+^ cells (18% at 16 weeks) or ALCAM^+^ cells (1.5% at 4 weeks). In addition, the percentage of 5-HT^+^ serotonergic neurons to total surviving cells at 12 weeks was 0.25±0.14%, which was smaller than that of CORIN^+^ cells (1% at 16 weeks) or ALCAM^+^ cells (0.3% at 4 weeks). These results suggest that the sorting of LRTM1^+^ cells is more effective for preparing DA neuron-enriched grafts.

In a transplantation of LRTM1^+^ cells (1.0 × 10^6^ cells in 4 μl) into the putamen of an MPTP-treated monkey, >2 × 10^5^ TH^+^FOXA2^+^ DA neurons survived at 3 months, which is a number adequate for expected improvement of Parkinsonian symptoms of the clinical patient^37^. In addition, these neurons extended TH^+^ neuronal fibres into the host brain and some of them were large in size and expressed DAT and GIRK2, suggesting mature A9 mDA neurons. Intriguingly, the largest number of TH^+^ cells that survived occurred when the sorted cells were injected after incubation for 2 weeks. This result may be due to the damage caused by the sorting procedure (day 15 cells and day 21 cells) or excessive differentiation towards mature DA neurons (day 35 cells). Optimization of the culture condition and culture duration is needed for better outcomes.

Another advantage of sorting LRTM1^+^ cells is the elimination of unwanted cells such as PSCs and uncommitted neural progenitors. A recent report showed that SOX1^+^PAX6^+^KI67^+^ early neural cells form rosettes in the grafts and contribute to graft expansion^21^. As shown in Fig. 4j,k,m,o,p, LRTM1^+^ cell-derived spheres on day 28 contained less NESTIN^+^ or SOX1^+^PAX6^+^KI67^+^ cells compared with those derived from unsorted or LRTM1^−^ cells. In addition, LRTM1^+^ cell-derived grafts in rat brain were smaller and contained less KI67^+^ cells (Fig. 5a–e,l,q). These results suggested that uncommitted neural progenitors (NESTIN^+^KI67^+^ and SOX1^+^PAX6^+^KI67^+^) might cause the large graft volume *in vivo*, and that the sorting of LRTM1^+^ cells reduces this risk.

In conclusion, a novel cell surface marker for mDA progenitors, LRTM1, can provide a powerful tool for efficient and safe cell therapy for PD patients. Although the current sorting yield (about 5%) is acceptable for clinical use, higher yields are preferred. New technologies, such as a more efficient DA differentiation protocol and a gentler and faster cell sorter, should help raise this percentage.

## Methods

### Production of LMX1A::GFP KI mESC line

The *Lmx1a-GFP* targeting vector was assembled using the ploxP-GFP-neo-DT-A vector that contains GFP complementary DNA and lox-P flanked HSV TK-Neomycin gene cassettes in a Bluescript SK^+^ (Stratagene) backbone. A 3.5 kb 5′-arm-containing genomic fragment just upstream to the initiation codon of *Lmx1a* and a 3.9 kb 3′-arm fragment were amplified by PCR and cloned separately into the NotI/SmaI and EcoRV sites of ploxP-GFP-neo-DT-A vector to generate the *Lmx1a*-targeting vector. LMX1A::GFP KI ESCs were generated by homologous recombination in 129SVEV ESC lines according to standard procedures and homologous recombination was confirmed by Southern blotting.

### Maintenance and neural differentiation of mESCs/iPSCs

mESC lines (EB5 (ref. 38); passages 35–45, LMX1A::GFP KI ESCs; passages 11-21, G4-2 (ref. 38); passages 20–30) and iPSC line (440A-3, a kind gift from Dr Okita, Kyoto University Center for iPS Cell Research and Application, Kyoto, Japan; passages 15–25) were maintained on mitotically inactivated mouse embryo fibroblast feeder layer in knockout DMEM medium supplemented with 1% penicillin/streptomycin (P/S; Gibco), 20% fetal bovine serum (Sigma-Aldrich), 0.1 mM 2-mercaptoethanol (2-ME; Wako), 2 mM L-glutamine (L-Glu; Sigma-Aldrich), 2,000 U ml^−1^ LIF (Merck Millipore) and 1 × Nucleosides (Merck Millipore). We changed the medium every day.

For neural induction, mESCs and miPSCs were replated in low cell adhesion 96-well plates (Lipidure-Coat Plate A-96U; NOF Corporation) at a density of 9,000 cells per well in a differentiation medium containing Glasgow minimum essential medium (GMEM) (Gibco) supplemented with 5% KSR, 0.1 mM MEM non-essential amino acids solution (Gibco), 2-ME, 1 mM sodium pyruvate solution (Pyruvate; Sigma-Aldrich) and 2 mM L-Glu. Moreover, we added both 100 ng ml^−1^ FGF8b (R&D) and SHH (R&D) to induce midbrain and FP cells, respectively, from day 1 to day 6. On day 7, we added 200 nM Ascorbic acid (AA; Nacalai), 20 ng ml^−1^ brain-derived neurotrophic factor (BDNF) (R&D), 1 × N-2 supplement (Gibco) and removed 5% KSR. We changed the medium every 2 days.

### Maintenance and neural differentiation of hESCs/iPSCs

This study was performed in conformity with ‘The Guidelines for Derivation and Utilization of Human Embryonic Stem Cells' of the Ministry of Education, Culture, Sports, Science and Technology of Japan, after approval by the institutional review board. hESCs (Kh-ES1 (ref. 19) passages 30–40) and hiPSCs (1039A1 (ref. 20) passages 15–25) were maintained on iMatrix-511(Nippi)-coated six-well plate at a density of 3 × 10^4^ cells per well with StemFit medium. When we began neural differentiation, these cells were dissociated into single cells with TrypLE select (Invitrogen) and were then replated on iMatrix-511-coated six-well plate at a density of 4 × 10^5^ cells per well with StemFit medium. Three days later, the medium was changed to a differentiation medium containing GMEM supplemented with 8% KSR, 0.1 mM non-essential amino acids solution, 2-ME, 1 mM Pyruvate and 2 mM L-Glu. In addition, 500 nM A83-01 (Wako) and 100 nM LDN193189 (STEMGENT) were added until day 7 and day 12, respectively; 2 μM Purmorphamine (Wako) and 100 ng ml^−1^ FGF8b (Wako) were added from day 1 to day 7; and 3 μM CHIR99021 (STEMGENT) was added from day 3 to day 12. We changed the medium every day^11^.

### Cell sorting and culture

To apply FACS, cultured cells were stained with anti-CORIN antibody (1:200) or anti-LRTM1 antibody (1:20; a kind gift from Dr Ono Y., KAN Research Institute, Japan) for 30 min and the cells were then stained with Alexa 647-conjugated goat anti-mouse IgG or Alexa 647-conjugated goat anti-rat IgG antibodies (1:400; Invitrogen), respectively. Dead cells were distinguished by 7-amino-actinomycin D (BD). FACS analysis was performed using FACS AriaII (BD Biosciences) and the data were analysed by FACSDiva software (BD Biosciences). About 10^8^ cells were applied to the sorting and ∼5 × 10^6^ LRTM1^+^ cells were obtained for the experiments.

The sorted cells were replated in low cell adhesion 96-well plates at a density of 2 × 10^4^ cells per well and were cultured as spheres in the following neural differentiation medium: for mouse cells from day 9 until day 11, DMEM/F12 supplemented with 1% P/S, 0.1 mM 2-ME, 200 nM AA, 2 mM L-Gln, 10 ng ml^−1^ glial cell-derived neurotrophic factor (GDNF), 20 ng ml^−1^ BDNF, 1 × N2 supplement and 1 × B-27 supplement; for human cells from day 14 until day 28, Neurobasal medium (Gibco) supplemented with 1% P/S, 0.1 mM 2-ME, 200 nM AA, 2 mM L-Gln, 400 mM dbcAMP (Sigma-Aldrich), 10 ng ml^−1^ GDNF, 20 ng ml^−1^ BDNF and 1 × B-27 supplement. We changed the medium every 3 days and 30 μM of Y-27632 (Wako) was added in the first medium. In the case of mouse cells, 5% KSR was also added from the day after cell sorting.

For *in vitro* studies of human LRTM1^+^ cells, the cultured cells were dissociated into single cells with Accumax (Innovative Cell Technologies) on day 28 and then replated on ornithine, laminin and fibronectin (OLF)-coated plates at a density of 2 × 10^5^ cells per cm^2^. The cells were incubated in a glia-conditioned medium consisting of Neurobasal medium supplemented with 1% P/S, 0.1 mM 2-ME, 200 nM AA, 2 mM L-Gln, 400 mM dbcAMP, 10 ng ml^−1^ GDNF, 20 ng ml^−1^ BDNF and 1 × B-27 supplement until day 70.

### Histological study of mouse embryonic brain

The experiments were performed according to the Guidelines for Animal Experiment of Kyoto University, the Guide for the Care and Use of Laboratory Animals of the Institute of Laboratory Animal Resources (Washington, DC, USA) and the Animal Research: Reporting *in vivo* Experiments (The ARRIVE guidelines)^39^. Pregnant C57BL/6N mice (*C57BL/6NCrSlc*; 10 weeks old) were obtained from Shimizu Laboratory Supplies (Kyoto, Japan) and killed with pentobarbital. The embryos were removed and their brains were cut with a cryostat (CM-1850; Leica Biosystems) at 20 μm thickness and attached onto the MAS-coated slide glasses (Matsunami, Osaka, Japan). The sections containing medial midbrain were chosen for the immunofluorescence study. The VM tissue was also used for the microarray and semi-quantitative RT–PCR analysis.

### Transplantation into the rat PD models

The experiments were performed according to the Guidelines for Animal Experiment of Kyoto University, the Guide for the Care and Use of Laboratory Animals of the Institute of Laboratory Animal Resources and the Animal Research: Reporting *In vivo* Experiments (The ARRIVE guidelines)^39^. Female SD rats (Sprague–Dawley; 9 weeks old) were obtained from Shimizu Laboratory Supplies (Kyoto, Japan). The PD models of SD rats were generated by injection of 6-OHDA (Sigma-Aldrich) into the medial forebrain bundle in the right side of the brain. The coordinates were calculated with reference to the bregma: anterior (*A*), −4.4 mm; lateral (*L*), −1.2 mm; ventral (*V*), −7.8 mm; and tooth bar (*TB*), −2.4 mm. A total of 13 μg of 6-OHDA was injected per rat in 2.5 μl of saline with 0.02% AA. In the case of miPSCs, LRTM1^+^ cells were sorted on day 9 and subjected to transplantation on day 11. The cultured spheres (∼1 × 10^5^ cells in 2 μl) in DMEM/F12 supplemented with 1% P/S, 0.1 mM 2-ME, 200 nM AA, 2 mM L-Gln, 10 ng ml^−1^ GDNF, 20 ng ml^−1^ BDNF, 1 × N2 supplement, 1 × B-27 supplement and 30 μM Y-27632 were injected stereotactically through a 22G needle into the right striatum (from the bregma: *A*, +1.0 mm; *L*, −3.0 mm; *V*, −5.0 mm and −4.0 mm; TB, 0 mm). A ROCK inhibitor, Y-27632, was used to reduce dissociation-related cell death during cell transplantation^40^,^41^. In the case of hiPSCs, LRTM1^+^ cells were sorted on day 14 and subjected to transplantation on day 28. The cultured spheres (∼1.3 × 10^5^ cells in 2 μl) in Neurobasal medium supplemented with 1% P/S, 0.1 mM 2-ME, 200 nM AA, 2 mM L-Gln, 400 mM dbcAMP, 10 ng ml^−1^ GDNF, 20 ng ml^−1^ BDNF and 1 × B-27 supplement, and 30 μM Y-27632 were injected stereotactically through a 22G needle into the right striatum. The rats received intraperitoneal injections of the immunosuppressant Cyclosporin A (Wako) every day starting 2 days before transplantation until the day of killing. Six, 12 or 16 weeks after transplantation, the animals were killed with pentobarbital and perfused with 4% paraformaldehyde (Wako). The brains were cut with a cryostat (CM-1850; Leica Biosystems) at 30 μm thickness and mounted. Every six sections containing the graft region were chosen for the immunofluorescence study. The graft volume was calculated by identifying SC-121 positive areas in every sixth 30 μm-thick section using a fluorescence microscope and the BZ-II Analyzer software program (BZ-9000; Keyence) and the total volume of the graft was determined according to Cavalieri's principle.

### Behavioural analysis

The methamphetamine-induced rotational behaviour was recorded for 90 min after intraperitoneal injection of methamphetamine (2.5 mg kg^−1^, Dainippon Sumitomo Pharma) and performed before and 4, 8, 12 or 16 weeks after transplantation. The apomorphine-induced rotational behaviour was recorded for 60 min after subcutaneous injection of apomorphine (0.1 mg kg^−1^, Wako) and performed before and 8 or 16 weeks after transplantation. The behaviour was automatically calculated by video-monitored rotational bowls.

### Transplantation into the non-human primate PD models

Two adult male cynomolgus monkeys (*Macaca fascicularis*; 4 years old) weighing 4.0–4.7 kg were provided by Shin Nippon Biomedical Laboratories (Kagoshima, Japan). The monkeys were cared for and handled according to Guidelines for Animal Experiments of Kyoto University. To generate a Parkinsonian model, the animals were given intravenous injection of MPTP HCl (0.4 mg kg^−1^ as a free base; Sigma-Aldrich) twice a week until signs of Parkinsonian symptoms, such as tremor, bradykinesia and impaired balance became evident^42^. The coordinates of the targets were obtained from magnetic resonance images. LRTM1^+^ cells were sorted on day 14 and subjected to transplantation on day 15, 21, 28 or 35. The cultured spheres in Neurobasal medium supplemented with 1% P/S, 0.1 mM 2-ME, 200 nM AA, 2 mM L-Gln, 400 mM dbcAMP, 10 ng ml^−1^ GDNF, 20 ng ml^−1^ BDNF and 1 × B-27 supplement, and 30 μM Y-27632 were injected stereotactically through a 22G needle into the right striatum along four tracts/side (∼1 × 10^6^ cells in 4 μl per 4 injection sites per tract). LRTM1^+^ cells at days 15 and 21 were injected into the right and left putamen of one monkey (two tracts for each condition), respectively. The cells at days 28 and 35 were injected the same way to another monkey. After surgery, the monkeys received antibiotics for 3 days and intramuscular injection of the immunosuppressant FK506 (0.05 mg kg^−1^, Astellas, Tokyo, Japan) until the day of sacrifice. Twelve weeks after transplantation, the animals were sacrificed and perfused with paraformaldehyde under deep anaesthesia. The brains were cut with a microtome (REM-710; YAMATO KOHKI Industrial Co., Ltd) at 40 μm thickness and mounted. Every 36 sections containing the graft region were chosen for the immunofluorescence study.

### Semi-quantitative RT–PCR and quantitative RT–PCR

Total RNA was extracted using an RNeasy Mini Kit or RNeasy Micro Kit (Qiagen) and cDNA was synthesized using Super Script III First-Strand Synthesis System (Invitrogen). Quantitative PCR reactions were performed with Ex Taq polymerase (Takara) or SYBR Premix Ex Taq (Takara), respectively, and with Thermal Cycler Dice Real Time System (Takara). The data were analysed using a delta-delta Ct method and normalized by glyceraldehyde 3-phosphate dehydrogenase levels. The primer sequences are shown in Supplementary Table 1.

### Dopamine release assay

hESC-derived sorted cells on day 28 were differentiated on an OLF-coated surface for 14 days, then washed with a low KCl solution (2.5 mM CaCl_2_, 11 mM glucose, 20 mM HEPES-NaOH, 4.7 mM KCl, 1.2 mM KH_2_PO_4_, 1.2 mM MgSO_4_ and 140 mM NaCl) and incubated in the low KCl solution for 2 min. The solution was subsequently replaced with a high KCl solution (2.5 mM CaCl_2_, 11 mM glucose, 20 mM HEPES-NaOH, 60 mM KCl, 1.2 mM KH_2_PO_4_, 1.2 mM MgSO_4_ and 85 mM NaCl) for 15 min. The solution was collected in centrifugal filters (Merck Millipore) and centrifuged for 1 min at 5,200 *g* to remove the debris. The concentration of dopamine was detected by HPLC using a reverse-phase column and an electrochemical detector system (HTEC-500; Eicom).

### Imnmunofluorescence studies

For *in vitro* studies, the cultured cells were incubated with PBS containing 2% Triton X-100 for 30 min and then blocked with PBS containing with 4% BlockAce (Megmilk Snow Brand Co., Ltd) and 0.1% Triton X-100 for 10 min. Before blocking, the antigen retrieval procedure including heating in a microwave oven for 5 min was used for anti-SOX1 and anti-PAX6 antibodies. The primary antibody reaction was performed overnight. For *in vivo* studies, the brain sections were stained likewise using the free-floating method. After reaction of the primary antibodies, the samples were stained with secondary antibodies conjugated with Alexa-488, -594 and -647 (1:400, Invitrogen) or Dylight-594 (1:200, Thermo Scientific) for 30 min and then stained with 200 ng ml^−1^ of 4′, 6′-diamidino-2-phenylindole. The primary antibodies are shown in Supplementary Table 2.

For 3,3′-diaminobenzidine (DAB) staining, the brain slices were incubated with PBS containing 30% H_2_O_2_ for 15 min and then incubated with primary antibody overnight. After rinsing, the samples were stained with biotinylated secondary antibody (Vector Labs) for 2 h. Finally, the samples were incubated with the ABC Elite Kit (Vector Labs).

Images were visualized with a fluorescence microscope (BZ-9000; Keyence) and a confocal laser microscope (Fluoview FV1000D; Olympus).

### Electrophysiological analysis

Whole-cell patch-clamp recordings were carried out on 70 day cultured hESC-derived LRTM1^+^ cells grown on an OLF-coated surface. The cells were treated with a physiological saline solution of the following composition: 2 mM CaCl_2_, 17 mM glucose, 2.5 mM KCl, 1 mM MgCl_2_, 125 mM NaCl, 26 mM NaHCO_3_ and 1.25 mM NaH_2_PO_4_. Patch pipettes (GC150TF-10, Clark) had a resistance of 3–4 MW when filled with an internal solution composed of 0.2 mM EGTA pH 7.3, 10 mM HEPES and 140 mM KCl. Recordings with a voltage clamp and current clamp were conducted with a patch-clamp amplifier (EPC-8, HEKA). The gigaseal resistances were in the range of 10–20 GW. The current signals were filtered at 5 kHz through a four-pole low-pass filter with Bessel characteristics (UF-BL2, NF), sampled with a 12-bit A/D converter and imported into a 32-bit computer (PC-9821Ra333, NEC). All experiments were carried out at room temperature.

### Microarray analysis

Total RNA was subjected to microarray analysis using an Ambion WT Expression Kit and Affymetrix GeneChip Whole Transcript (WT) Expression Arrays (Ambion, Life Technologies). Comparisons were performed between day 9 mESC-derived CORIN^+^LMX1A::GFP^+^ and CORIN^−^LMX1A::GFP^+^ cells and between E11.5 fetal mouse VM-derived CORIN^+^ and CORIN^−^ cells. The data were analysed using the GeneSpring software programme (version 13.0; Agilent Technologies).

### Statistical analysis

Statistical significance between two samples was determined with Student's *t*-test (GraphPad Prism 5; GraphPad). The statistical significance among multiple samples was determined with one-way analysis of variance with Bonferroni's multiple comparison tests or two-way analysis of variance with Dunnett's multiple comparisons tests. The data were considered statistically significant for *P*<0.05 and are shown as the mean±s.e. (s.e.m). All data were acquired from at least three independent experiments.

### Data availability

Microarray data have been deposited in the NCBI Gene Expression Omnibus database under accession code GSE72875. The authors declare that all data supporting the findings of this study are available within the article and its Supplementary Information files or from the corresponding author upon reasonable request.

## Additional information

**How to cite this article**: Samata, B. *et al*. Purification of functional human ES and iPSC-derived midbrain dopaminergic progenitors using LRTM1. *Nat. Commun.* **7**, 13097 doi: 10.1038/ncomms13097 (2016).

## Supplementary Material

Supplementary Information Supplementary Figures 1-10, Supplementary Tables 1, 2

Supplementary Data 1 Gene expression array data of E11.5 mouse and day 9 mouse ESCs
