# Supplementary material for: Purification of functional human ES and iPSC-derived midbrain dopaminergic progenitors using LRTM1
Source: Nat Commun. 2016 Oct 14;7:13097. doi: 10.1038/ncomms13097 (PMC5067526; doi:10.1038/ncomms13097)
Supplement: Supplementary Information — Supplementary Figures 1-10, Supplementary Tables 1, 2 [file ncomms13097-s1.pdf]

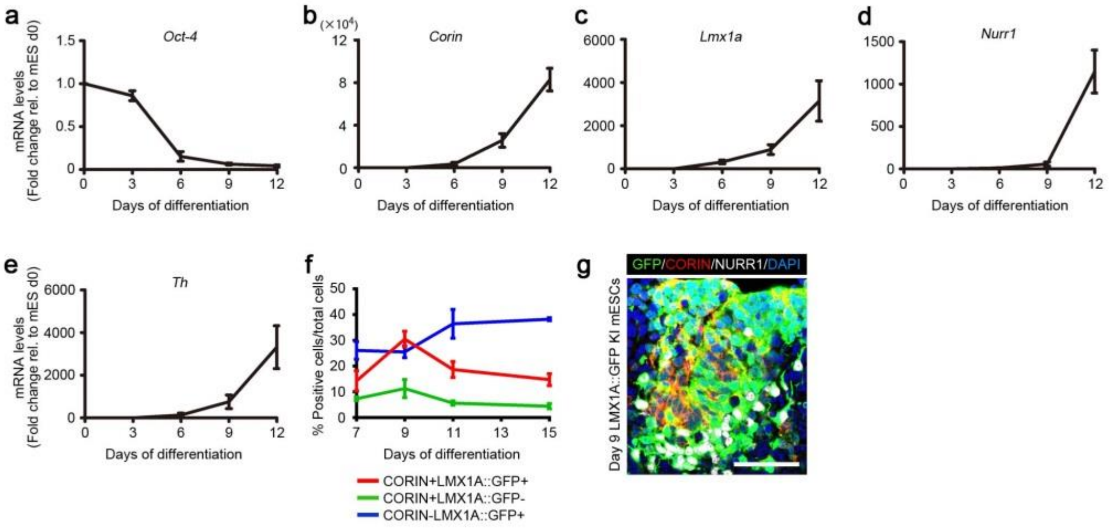

Supplementary Figure 1 Induction of DA neurons by the SFEBq method. (**a-e**) Gene expression analysis for pluripotent (*Oct-4*; **a**), floor plate (*Corin*; **b**), DA progenitor (*Lmx1a* and *Nurr1*; **c,d**) and DA neuron (*Th*; **e**) markers by quantitative RT-PCR (n = 4). (**f**) Temporal protein expression of CORIN and LMX1A::GFP (n = 3). Error bars indicate s.e.m. (**g**) Immunofluorescence image of LMX1A KI mouse ESCs for DAPI (blue), LMX1A::GFP (green), CORIN (red) and NURR1 (white) on day 9. Scale bar, 50 μm.

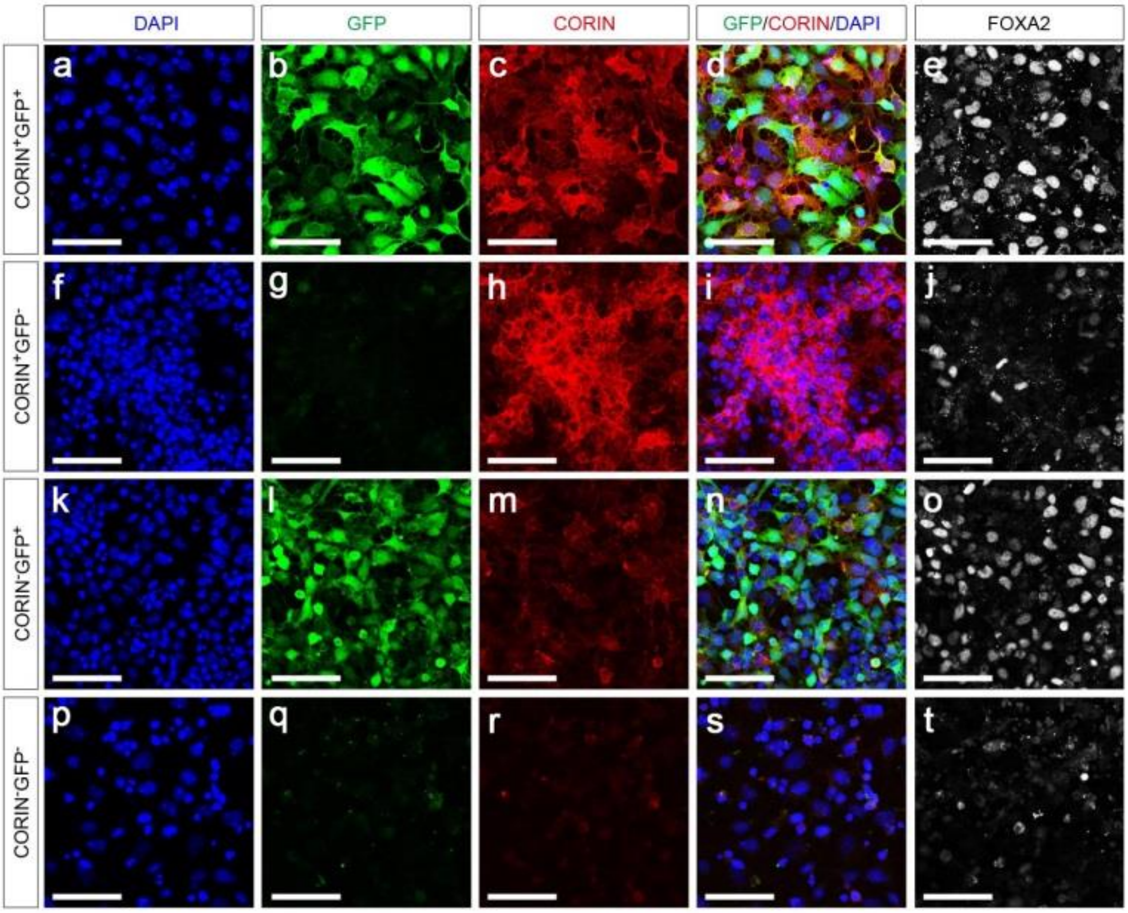

Supplementary Figure 2 Purification of DA progenitors by co-expression of CORIN and LMX1A::GFP. (**a-t**) Immunofluorescence images of sorted cells on day 9 for DAPI (blue), LMX1A::GFP (green), CORIN (red) and FOXA2 (white). Scale bars, 50 μm.

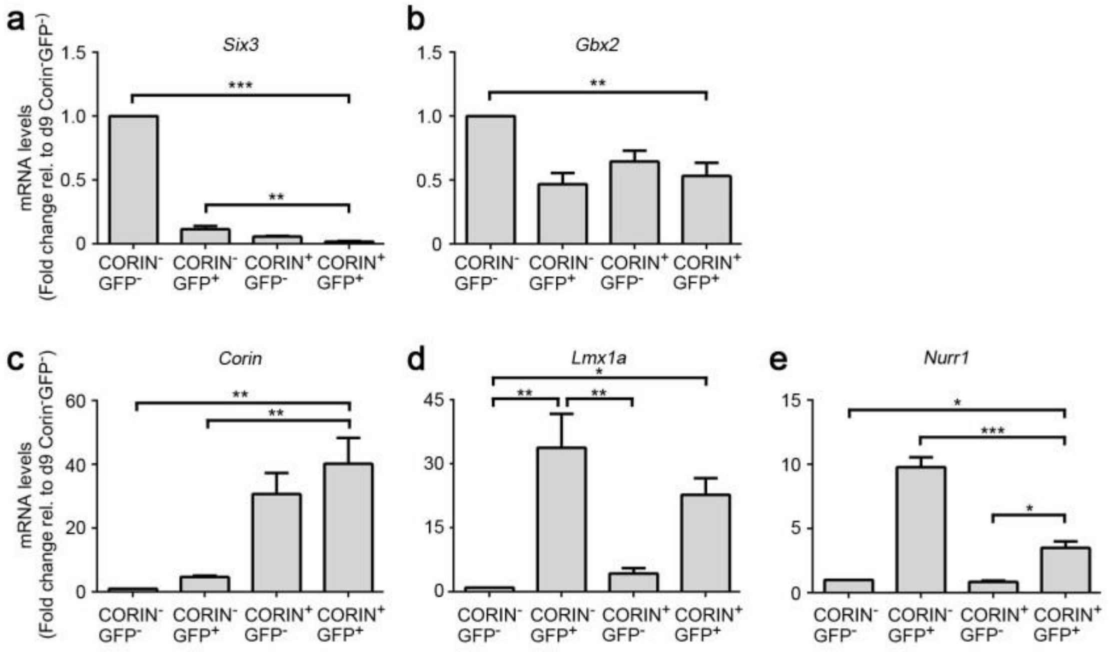

Supplementary Figure 3 Gene expression analysis of FACS-purified cells. Gene expression analysis of the sorted cells for forebrain (*Six3*; **a**), hindbrain (*Gbx2*; **b**), floor plate (*Corin*; **c**) and DA progenitor (*Lmx1a* and *Nurr1*; **d,e**) markers on day 9 by quantitative RT-PCR ($n = 4$). Asterisks indicate statistical significance as determined by a one-way ANOVA with Bonferroni's multiple comparison test; *, $P < 0.05$, **, $P < 0.01$ and ***, $P < 0.001$. Error bars indicate s.e.m.

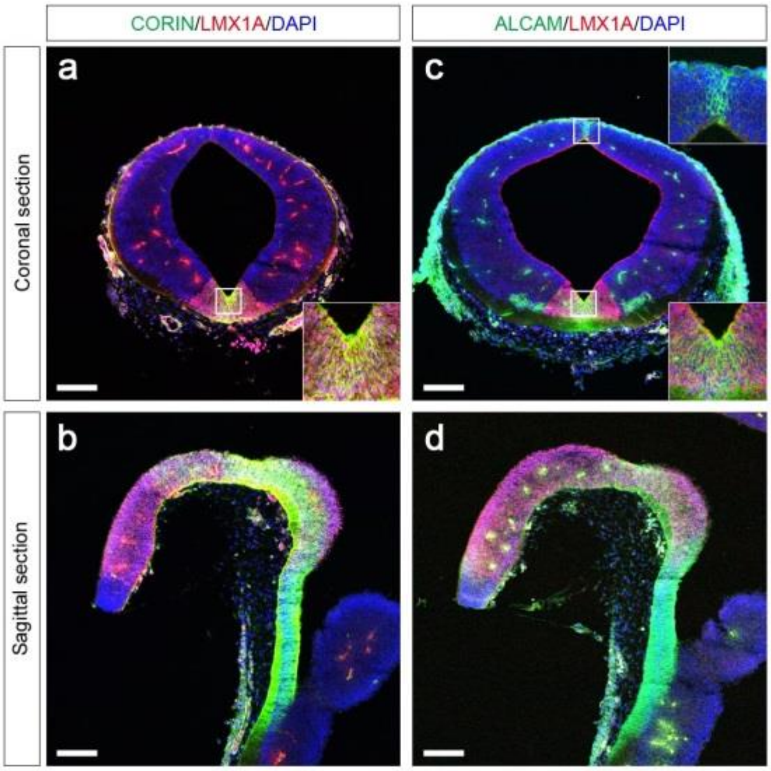

Supplementary Figure 4 CORIN and ALCAM expression in developing mouse brain. (**a-d**) Immunohistochemical images of E11.5 fetal mouse brain for CORIN (green), ALCAM (green), LMX1A (red) and DAPI (blue) in the coronal section (**a,c**) and in the sagittal section (**b,d**). Insets indicate magnified images of CORIN^{+} or ALCAM^{+} cells. Scale bars, 100 μm.

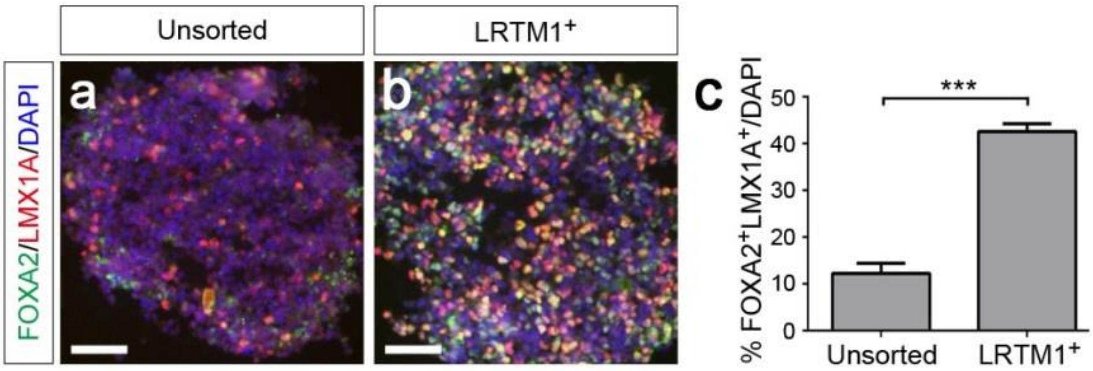

Supplementary Figure 5 Purification of mDA progenitors from fetal mouse VM using LRTM1. (**a,b**) Immunofluorescence images of E11.5 fetal mouse VM-derived unsorted cells (**a**) and LRTM1$^+$ cells (**b**) for FOXA2 (green), LMX1A (red) and DAPI (blue). (**c**) Quantification of FOXA2$^+$LMX1A$^+$ cells in unsorted cells vs. LRTM1$^+$ cells (n = 4). Asterisks indicate statistical significance as determined by student's t-test; ***, $P < 0.001$. Scale bars, 50 µm. Error bars indicate s.e.m.

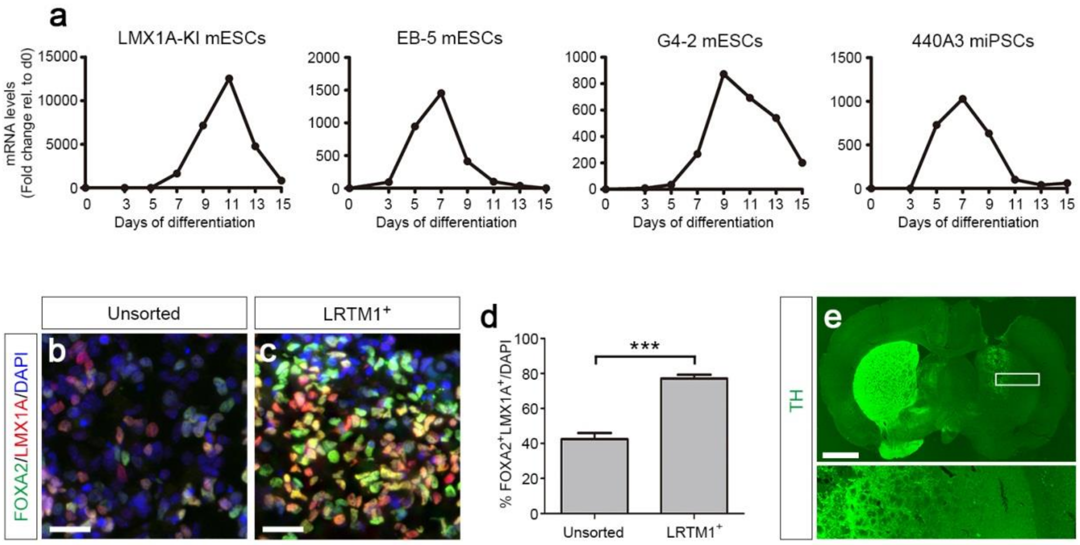

Supplementary Figure 6 Purification of mDA progenitors from mouse iPSCs using LRTM1. (a) Gene expression analysis of mouse ESC lines (LMX1A::GFP, EB5, G4-2) and iPSC line (440A3) for Lrtm1 by quantitative RT-PCR. **(b,c)** Immunofluorescence images of spheres derived from unsorted cells and LRTM1^{+} cells for FOXA2 (green), LMX1A (red) and DAPI (blue) on day 9. Scale bars 100 μm. **(d)** Quantification of FOXA2^{+}LMX1A^{+} cells in unsorted cells (n = 6) versus LRTM1^{+} cells (n = 5). Asterisks indicate statistical significance as determined by student's t-test, ***, $P < 0.001$. Error bars indicate s.e.m. **(e)** Immunohistochemical image of mouse iPSC-derived LRTM1^{+} graft for TH (green) (n = 3). Scale bars, 2 mm.

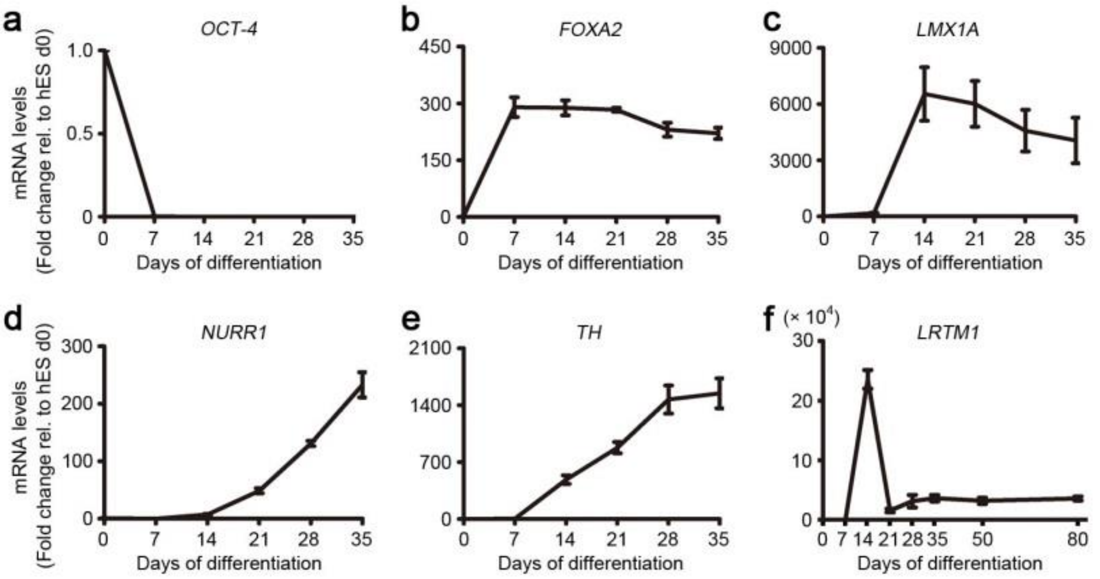

Supplementary Figure 7 Induction of DA neurons from human ESCs by adhesive culture. (**a-e**) Gene expression analysis for pluripotent (*OCT-4*; **a**), floor plate (*FOXA2*; **b**), DA progenitor (*LMX1A* and *NURR1*; **c,d**) and DA neuron (*TH*; **e**) markers by quantitative RT-PCR (n = 4). (**f**) LRTM1 mRNA expression showed a peak at 14 days after differentiation (n = 3). Error bars indicate s.e.m.

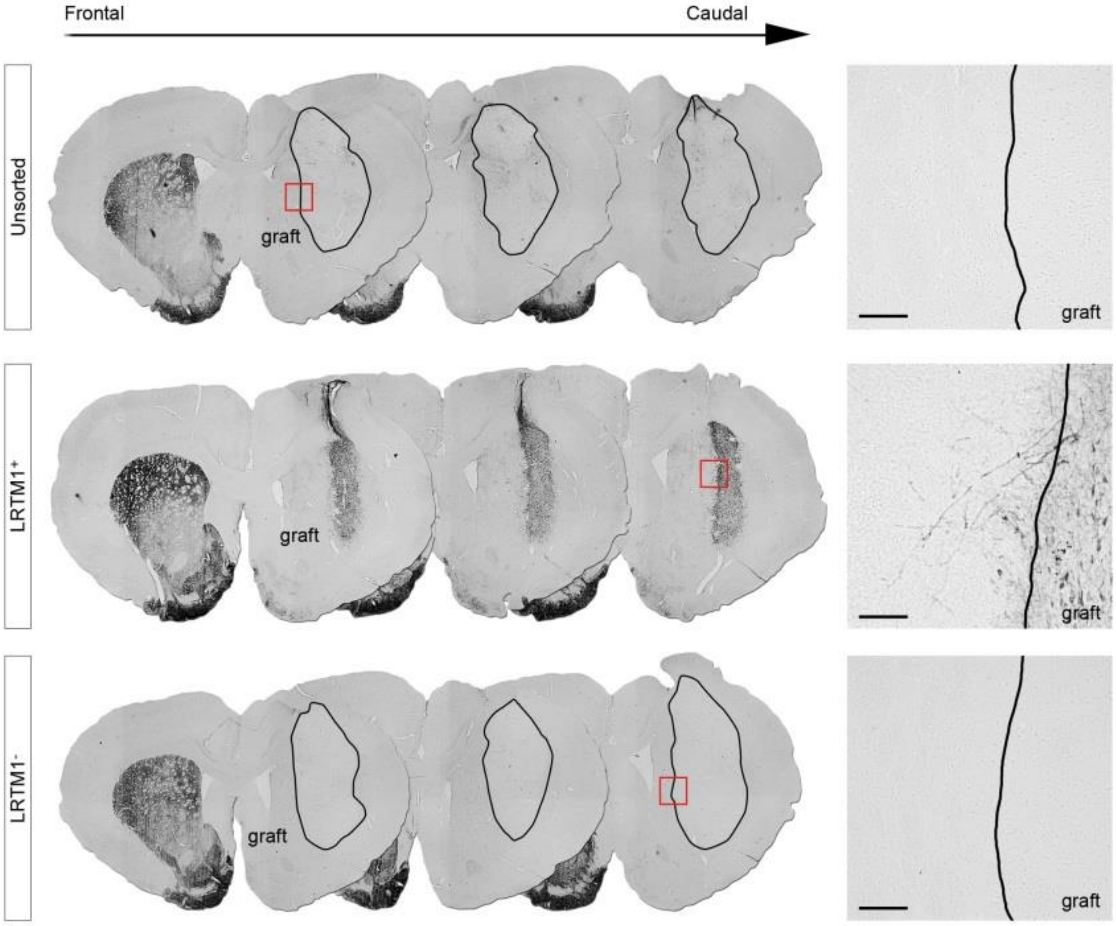

Supplementary Figure 8 In vivo survival of FACS sorted human iPSCs. TH-DAB staining of the sequential brain slices transplanted with unsorted cells, LRTM1^{+} cells and LRTM1^{-} cells at 12 weeks after transplantation. The outline indicates grafts from unsorted cells and LRTM1^{-} cells. Red squares indicate magnified images of the grafts. Scale bars, 100 μm.

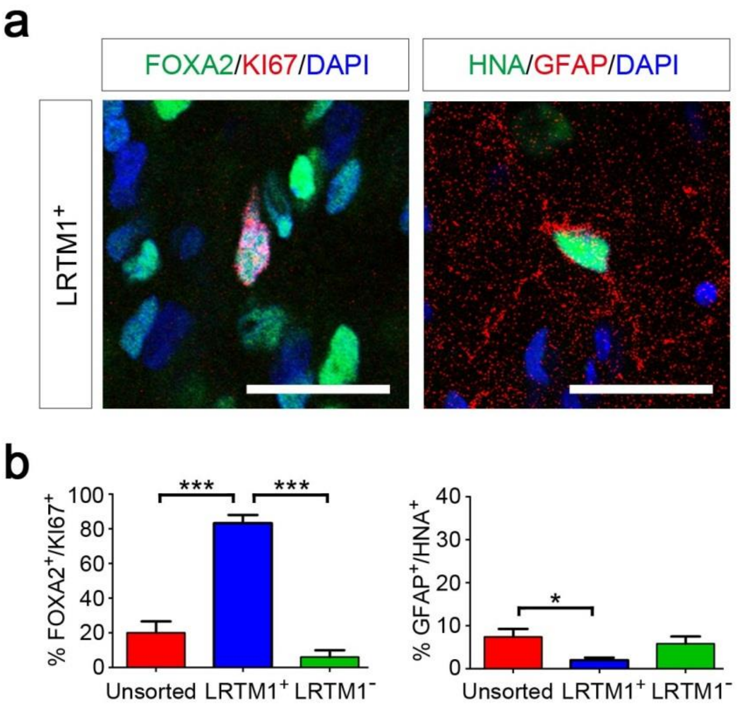

Supplementary Figure 9 Donor-derived DA progenitors and astrocytes in the graft at 12 weeks after transplantation. (**a**) Immunohistochemical images of the graft derived from unsorted cells, LRTM1$^+$ cells and LRTM1$^-$ cells for FOXA2 (green), HNA (green), KI67 (red), GFAP (red) and DAPI (blue) at 12 weeks. Scale bar, 25 μm. (**b**) Quantification of FOXA2$^+$ cells per KI67$^+$ cells and GFAP$^+$ cells per HNA$^+$ surviving human cells in unsorted cells (n = 7) vs. LRTM1$^+$ cells (n = 12) vs. LRTM1$^-$ cells (n = 5). Asterisks indicate statistical significances as determined by a one-way ANOVA with Bonferroni's multiple comparison test; *, $P < 0.05$, and ***, $P < 0.001$. Error bars indicate s.e.m.

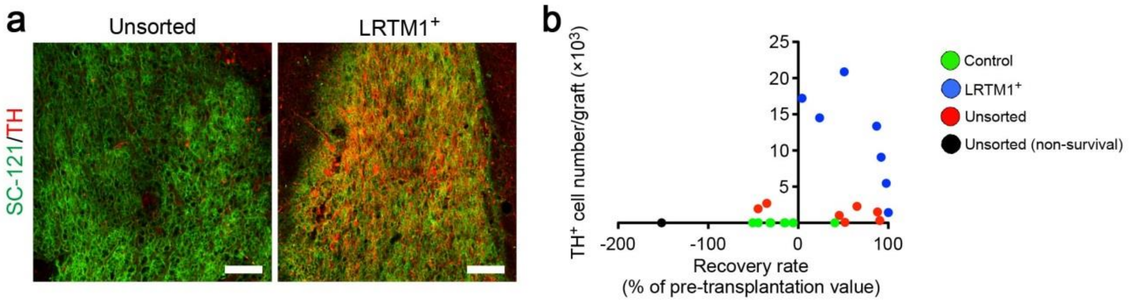

Supplementary Figure 10 In vivo study of human iPSC-derived DA neurons. (**a**) Immunohistochemical images of the graft derived from unsorted cells and LRTM1$^+$ cells for SC-121 (green) and TH (red) at 16 weeks. Scale bar, 100 μm. (**b**) Correlation between the TH$^+$ cell number and the recovery rate (pre- vs. 16 weeks post-transplantation) in a methamphetamine-induced rotational analysis. control: n = 8; LRTM1$^+$ cells: n = 7; unsorted cells: n = 7.

Supplementary Table 1 Lists of primers for semi-quantitative RT-PCR (S) and quantitative RT-PCR (Q).

Gene name	Types of primer	Forward primer	Reverse primer
mCorin	S	GAGGCTGCAAAAGAAGGATG	TGTACAAGGGCAGGGAAAAG
	Q	CCCTTCTGTGCTCCAAGCAAGACTG	AGAGCACTGCCACGGCCACC
mGapdh	S	GCAGTGGCAAAGTGGAGATT	ATGTAGGCCATGAGGTCCAC
	Q	CCGCCTGGAGAAACCTGCCAAGT	GGGAGTTGCTGTTGAAGTCGCAGG
mLrtm1	S	ACCCTTGGATTTGTGACTGC	CCTTGGTACTGGGCTGTGAT
	Q	TGTTGAATGAGGGTTTGTGCT	TCCACGGAGTTTGATGATGG
mAnxa2	Q	CATTCTACACCCCCAAGTGC	CAGCTTTCGGAAGTCTCCAG
mFolr1	Q	GGCCCTGAGGACAATTTACA	CCCAGAGCAGCACTAAGGAC
mOtx2	Q	CTCGACGTTCTGGAAGCTCT	CTGGGTGGAAAGAGAAGCTG
mTacr1	Q	GTGCAACCTACCTGGCAAAT	TGACCTTGTACACGCTGCTC
mTm4sf1	Q	GCTGCGGCTACGAAAACTAC	GCCTCCAAGCATTCCATTTA
mGbx2	Q	GCAAGGGAAAGACGAGTCAAA	GGCAAATTGTCATCTGAGCTGTA
mLmx1a	Q	GTCCCAGAACCATCCTGACC	GGAACCACACCTGAACCACA
mNestin	Q	CAGCAACTGGCACACCTCAA	GCCAAGAGAAGCCTGGGAAC
mNurr1	Q	GGCTCCCTTCACAACTTCCA	AGAGTGACAGGCGGGAGACA
mOct3/4	Q	AGATCACTCACATCGCCAATC	GGAAAGGTGTCCCTGTAGCC
mPax7	Q	GTGGAATCAGAACCCGACCTC	GTAGTGGGTCCTCTCGAAGGC
mSix3	Q	CCGGAAGAGTTGTCCATGTTC	CGACTCGTGTTTGTTGATGGC
mTh	Q	CAGTGCCAGAGAGGACAAGG	AGGTCCAGGTCAGGGTCAAA
hFOXA2	Q	TTCAGGCCCGGCTAACTCT	AGTCTCGACCCCCACTTGCT
hGAPDH	Q	ATGGGGAAGGTGAAGGTCG	TAAAAGCAGCCCTGGTGACC

hLMX1A	Q	TGCCTGGAGACCACATGCA	TCGCTATCCAGGTCATGGAAA
hLRTM1	Q	ATTGCCACTTGCTCGGTCTT	TCCTTTCCCTTCCAGGTGTCT
hNURR1	Q	CGAAACCGAAGAGCCCACAGGA	GGTCATAGCCGGGTTGGAGTCG
hOCT3/4	Q	AGACCATCTGCCGCTTTGAG	GCAAGGGCCGCAGCTT
hTH	Q	GCAGTTCTCGCAGGACATTG	CGGCACCATAGGCCTTCA

Supplementary Table 2 List of Antibodies and Dilutions, Related to Experimental Procedures.

Protein	Species	Company	Dilution
ALCAM	Goat	R&D Systems	1:200
CALBINDIN	Mouse	Swant Swiss Antibodies	1:1,000
CORIN	Mouse	Donated by the KAN laboratory	1:200
DAT	Rat	Abcam	1:200
FOXA2	Goat	R&D Systems	1:500
GFP	Rabbit	MBL Co., LTD.	1:1,000
GIRK2	Rabbit	Alomone Labs	1:200
Ki67	Rabbit	Novocastra	1:1,000
LMX1A	Hamster	Donated by the KAN laboratory	1:200
Lrtm1	Rat	Donated by the KAN laboratory	1:20
HNA	Mouse	Merck Millipore	1:1,000
NURR1	Rabbit	Santa Cruz Biotechnology, Inc.	1:200
NURR1	Rat	Donated by the KAN laboratory	1:1,000
PAX6	Mouse	BD Biosciences	1:200
PITX3	Rabbit	Merck Millipore	1:200
SOX1	Goat	R&D Systems	1:100
STEM121	Mouse	StemCells, Inc.	1:1,000
TH	Rabbit	Merck Millipore	1:1,000
TH	Sheep	Merck Millipore	1:1,000
TUJ1 (also known as Tubb3)	Mouse	Covance	1:2,000
